# Characterization of nanosensitive multifractality in submicron scale tissue morphology and its alteration in tumor progression

**DOI:** 10.1117/1.JBO.26.1.016003

**Published:** 2021-01-11

**Authors:** Nandan Das, Sergey Alexandrov, Katie E. Gilligan, Róisín M. Dwyer, Rolf B. Saager, Nirmalya Ghosh, Martin Leahy

**Affiliations:** a National University of Ireland, Tissue Optics and Microcirculation Imaging, Galway, Ireland; b Linköping University, Biomedical Imaging and Spectroscopy, Clinical Instrument Translation, Linköping, Sweden; c National University of Ireland Galway, Discipline of Surgery, Lambe Institute for Translational Research, Galway, Ireland; d Indian Institute of Science Education and Research Kolkata, Bio-Optics and Nano-Photonics, Kolkata, India; e Institute of Photonic Sciences, Barcelona, Spain

**Keywords:** spectroscopy, optical coherence tomography, submicron scale self-similarity, nanosensitive multifractality, early disease detection, cancer, tumor

## Abstract

**Significance:** Assessment of disease using optical coherence tomography is an actively investigated problem, owing to many unresolved challenges in early disease detection, diagnosis, and treatment response monitoring. The early manifestation of disease or precancer is typically associated with subtle alterations in the tissue dielectric and ultrastructural morphology. In addition, biological tissue is known to have ultrastructural multifractality.

**Aim:** Detection and characterization of nanosensitive structural morphology and multifractality in the tissue submicron structure. Quantification of nanosensitive multifractality and its alteration in progression of tumor.

**Approach:** We have developed a label free nanosensitive multifractal detrended fluctuation analysis(nsMFDFA) technique in combination with multifractal analysis and nanosensitive optical coherence tomography (nsOCT). The proposed method deployed for extraction and quantification of nanosensitive multifractal parameters in mammary fat pad (MFP).

**Results:** Initially, the nsOCT approach is numerically validated on synthetic submicron axial structures. The nsOCT technique was applied to pathologically characterized MFP of murine breast tissue to extract depth-resolved nanosensitive submicron structures. Subsequently, two-dimensional MFDFA were deployed on submicron structural *en face* images to extract nanosensitive tissue multifractality. We found that nanosensitive multifractality increases in transition from healthy to tumor.

**Conclusions:** This method for extraction of nanosensitive tissue multifractality promises to provide a noninvasive diagnostic tool for early disease detection and monitoring treatment response. The novel ability to delineate the dominant submicron scale nanosensitive multifractal properties may also prove useful for characterizing a wide variety of complex scattering media of non-biological origin.

## 1 Introduction

Early disease progression in living tissues expected to exhibit nanosensitive structural alteration at the submicron scale. It is highly desirable to develop noninvasive, label-free techniques to detect nanoscale changes in biological tissue for early diagnosis and better treatment. Recently, many optical nanoscopic techniques were developed based on labeling^1^^–^^6^ and are limited to superficial imaging.^7^^–^^13^ It is a challenging task for researchers to develop a diagnostic system that can provide label-free depth-resolved detection. There are few early developments that demonstrated averaged nanosensitive structural detection over a volume- rather than depth-resolved detection.^14^^,^^15^ These methods can identify overall nanosensitive changes rather than depth-resolved alteration, which is crucial to visualize subtle changes of local submicron structure for better diagnosis. In this regard, our research group actively engaged to develop nanosensitive optical coherence tomography (nsOCT) to detect depth-resolved submicron scale structure with few nanometer accuracy.^16^^–^^18^ We have recently demonstrated label-free nsOCT-based imaging technique to visualize few nanometer structural changes^19^^,^^20^ and its application in cornea crosslinking,^21^ and wound healing study.^22^ There is a recently demonstrated application of nsOCT for *in vivo* detection of nanosensitive changes of the human tympanic membrane in otitis media.^23^ In addition, biological tissue is known to have submicron structural multifractality.^24^^–^^28^ Although, these studies are based on superficial detection and do not provide underlying tissue multifractality. Multifractality is a special class of self-similarity where multiple scaling exponents (generalized Hurst exponents) are extracted to quantify existing multifractality in a complex system.^29^^,^^30^ For both the fundamental study of biological processes and early diagnosis of pathological processes, detection of multifractality in depth-resolved nanosensitive tissue submicron structural morphology is important. Therefore, we have developed a label-free nanosensitive multifractal detrended fluctuation analysis (nsMFDFA) technique in combination with multifractal analysis and nsOCT to extract nanosensitive multifractal parameters in biological tissue. Recently, we have numerically and experimentally validated nsOCT approach on synthetic submicron axial structure with few nanometer accuracy.^20^ Here we have numerically validated our proposed nanosensitive multifractal analysis approach in combination of nsOCT simulation and multifractal analysis in a tissue-like randomized synthetic phantom. This approach demonstrated its applicability to measure depth-resolved nanosensitive multifractality in submicron structure in biological tissue. After successful validation, we have applied nsOCT method to construct depth-resolved *en face* images of dominant submicron structure with nanometer scale sensitivity in murine MFP. Subsequently, we have deployed two-dimensional multifractal detrended fluctuation analysis (2D-MFDFA)^31^^–^^33^ approach to extract depth-resolved nanosensitive multifractality in MFP. In an initial *ex vivo* study on murine tissue, we found interesting change in depth-resolved nanosensitive multifractality in submicron structures after tumor formation in breast tissue samples. This method for extraction of nanosensitive tissue multifractality promises to develop a noninvasive diagnosis tool for the detection of cancer development. This newly developed method offers exciting depth-resolved ultrastructural detection for better treatment and monitoring if there is a tumor response to treatment.

## 2 Materials and Methods

### 2.1 Nanosensitive Optical Coherence Tomography

**Fig. 1:**
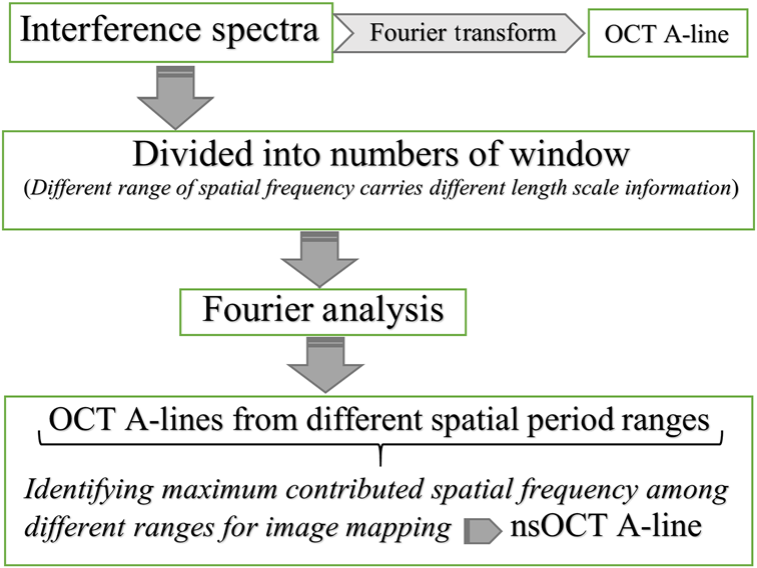
Flowchart: construction of nsOCT.

Flowchart of nsOCT is shown in Fig. 1. Recorded interference spectra divided into number of windows before applying Fourier transform for nsOCT construction. Then identifying spatial frequency corresponding to maximum contributed spectral window at different depths of constructed A-line. Subsequently, identified maximum spatial periods map as nsOCT at different depths. In nsOCT approach, detectable spatial period (Hz=λ/2  n; n = refractive index of the medium) depends on wavelength range of the broadband source (λ=1176 to 1413 nm).^18^ Therefore, in case of biological tissue, we can detect axial structure range from 420 to 504 nm with few nanometer accuracy.^18^ It is worth mentioning here that biological tissue may have axial structures ranging from 0 to ∞ (or 0 to tissue physical size). However, our nsOCT approach has a limitation of detection from 420 to 504 nm, which is limited by wavelength range and finding tissue structural differences based on this specified axial scale range only. There may be other axial structures that also exist in biological tissue beyond our detection range that need further study with a much more sophisticated system having a higher wavelength band. In this direction, our research group developing new ultra-high wavelength band nsOCT system.

### 2.2 Numerical Simulations for Nanosensitive Multifractal Detrended Fluctuation Analysis in Randomized Submicron Structure

In our recent publications,^20^^,^^34^ we have experimentally and numerically demonstrated the implementation of nsOCT to image a commercially fabricated sample with 431.56 nm axial periodic structures. In Ref. 34, we have demonstrated that we can detect random submicron dominant structures at different depths with ∼5  nm accuracy. In this study, we have numerically synthesized randomized 3D structure with submicron scale axial structure varies from 445 to 485 nm. The dimension of synthetic volume is 1024×1024×400  voxels. We have followed standard Fourier domain OCT theory and simulation strategy^35^^,^^36^ to construct nsOCT. The MATLAB 2019b (MathWorks^®^) has been used for implementation of simulation. The OCT signal was constructed as an interference spectrum of the reflected light from different layers of synthetic sample with the reflected light from a gold mirror. We have also added suitable noise in detector and source spectra to mimic experimental reality. The signal-to-noise ratio was 86 dB in this simulation. The inverse Fourier transform was performed to form A-lines nsOCT of a synthetic volume as each lateral position of 1024×1024  pixels. Then we have performed 2D-MFDFA on synthetic and nsOCT constructed *en face* images to compare.

### 2.3 Multifractal Analysis on Nanosensitive Submicron Structural En Face Images

We have followed our established nsOCT methodology to construct depth-resolved dominant structures of synthetic and tissue volume. Flowchart of nsOCT processing displayed in Fig. 1. Subsequently, we have applied 2D-MFDFA on nsOCT constructed *en face* images to extract nanosensitive multifractal parameters namely, Hurst exponent [h(q=2)] or correlation and width of singularity spectra (Δα) or strength of multifractality. To study the hidden nanosensitive multifractal properties in the submicron scale surface morphology, a 2D-MFDFA^31^^–^^33^ is implemented here. This method is simple yet innovative and has easy computer implementation. This proposed method is the modification of one-dimensional MFDFA, which is implemented to study the multiple scaling exponents of one-dimensional signals and for identification of long-range correlations in non-stationary time series.^30^ The detailed 2D-MFDFA approach can be found in Ref. 31. We have briefly discussed about 2D-MFDFA steps here.

Step 1: The two-dimensional *en face* nsOCT image [size: M(=500  μm]×N(=500  μm)) divided into non-overlapping square subsurface $M_{s}\times N_{s}$ of equal length s, where $M_{s}\equiv [M/s]$ and $N_{s}\equiv [N/s]$ are positive integers. Each square segment denoted by $X_{m,n}$, where $m=1,2,\ldots ,M_{s}$ and $n=1,2,\ldots ,N_{s}$. Here size of square subsurface s varies from 4 to 32.
Step 2: Each square segment $X_{m,n}$ is cropped and denoted as $G_{m,n}(i,j)$, where, i,j=1,2,…,s.
Step 3: The local fit ${\overset{˜}{G}}_{m,n}(i,j)$ for each $G_{m,n}(i,j)$ is calculated by fitting it with a bivariate polynomial function as
  $${\tilde{G}}_{m,n}(i,j)=ai+bj+c,$$ (1)
  where a, b, and c are free parameters and determined by the least square fitting in nanosensitive subsurface $G_{m,n}$ at different locations i,j=1,2,…,s in constructed *en face* nsOCT. The residual nanosensitive axial size variation or detrended subsurface is given by $y_{m,n}(i,j)$ at different location as i,j=1,2,…,s as
  $$y_{m,n}(i,j)=G_{m,n}(i,j)-{\tilde{G}}_{m,n}(i,j).$$ (2)
Step 4: The detrended fluctuation function F(m,n,s) for the segment $X_{m,n}$ is defined as
  $$F^{2}(m,n,s)=\frac{1}{s^{2}}\overset{s}{\underset{i=1}{\sum }}\sum_{j=1}^{s}y_{m,n}{\left(i,j\right)}^{2}.$$ (3)
  Here $s^{2}$ is the number of pixels in segmented square with size s.
  The q’th order fluctuation function for a nsOCT mapped *en face* image at each depth is
  $$F_{q}(s)=\{\frac{1}{M_{s}N_{s}}\sum_{m=1}^{M_{s}}\sum_{n=1}^{N_{s}}{\left[F(m,n,s)\right]}^{q}{\}}^{1/q}.$$ (4)
  Here $M_{s}N_{s}$ is the total number of segmented square surface in each *en face* image with size s. The q is order of moments varies from −3 to +3 with 0.5 interval. At q=2, the above fluctuation function represents variance of the *en face* nsOCT map. Note that, in principle, we can calculate generalized Hurst exponents for q=−∞ to +∞. But here most of variation of h(q) happening within q=−3 to +3. Therefore, we have not extended analysis for other q values which does not provide significance multifractality [variation of h(q)] and are computationally expensive.
Step 5: The generalized Hurst exponents [h(q)] can be extracted for multiple order of moments (q=−3:0.5:+3) by considering long-range power law behavior of this calculated fluctuation function $F_{q}(s)$ as
  $$F_{q}(s)\propto s^{h(q)}.$$ (5)
  Here in this present study, we have found detected nanosensitive structure follow power law behavior over length scale range s=4 to 32. Therefore, values of scale s optimize to varies from 4 pixel to 32 pixels (8 to 64  μm) in this nsMFDFA analysis to extract mutifractality.
  From this above equation, the scaling exponent h(q) is obtained by calculating slopes of linear fitting on $\ln\;F_{q}(s)$ versus log s plots. The h(q) is known as the generalized Hurst exponents and H=h(q=2) is called the Hurst index of the *en face* nsOCT surface.

The classical multifractal scaling exponent τ(q) corresponding to every q value is given by

$$\tau (q)=qh(q)-D_{f}=qh(q)-2,$$ (6)

where $D_{f}$ is the fractal dimension of the geometric support of the multifractal measure and $D_{f}=2$ in this study.

The two-scaling exponent h(q) and τ(q) along with singularity spectrum f(α) can completely characterize any multifractal surface. The singularity spectrum f(α), which characterizes the singularity strength or multifractality of *en face* nsOCT surface is related to τ(q) via a Legendre transformation as the Holder exponents,

$$\alpha (q)=\tau^{\prime }(q)=h(q)+qh^{\prime }(q),$$ (7)

and the singularity spectrum,

f(α)=qα(q)−τ(q)=q[α−h(q)]+2. (8)

Here f(α) measures global singularity and α(q) characterizes the local singularity of the *en face* image. The width of the singularity spectrum f(α) as a measure of multifractality strength as

$$\Delta \alpha =\alpha_{\max}-\alpha_{\min},$$ (9)

where $\alpha_{\max}=\max\{\alpha (q),q\in [-3,3]\}$ and $\alpha_{\min}=\min\{\alpha (q),q\in [-3,3]\}$. Δα measures the nanosensitive submicron scale multifractality at each *en face* images. The higher value of Δα in the submicron scale *en face* indicates higher strength of multifractality. The Hurst scaling exponents: h(q=2)=0.5, >0.5, and <0.5 correspond to uncorrelated, long-range correlated, and anti-correlated fluctuations, respectively, in nanosensitive *en face* images at different depths. The h(q=2)∈(0,1).

In this study, we have characterized (a) Hurst exponent [h(q=2)] and (b) width of the singularity spectrum f(α), (Δα) to measure correlation and multifractality, respectively, on detected nanosensitive submicron scale *en face* images.

### 2.4 Tissue Sample Preparation

All animal procedures were performed in accordance with the Guidelines for Care and Use of Laboratory Animals of the “Animal Care Research Ethics Committee (ACREC), National University of Ireland Galway (NUIG)” and approved by the “Health Product Regularity Authority (HPRA), Ireland”.

Female BALB/c mice (Charles River Laboratories Ltd.) aged between 6 and 8 weeks were employed. A mouse received a mammary fat pad (MFP, 4th inguinal) injection of $1\times 10^{5}$ 4T1 breast cancer cells suspended in 100  μl RPMI medium. The early stage tumor was detected by palpation after seven days of injection and was visually inspected. Tumor growth was monitored using calipers measurement. The tumor size was $715\;{\mathrm{mm}}^{3}$. Animals were sacrificed by ${\mathrm{CO}}_{2}$ inhalation. Tumor tissue and healthy portion were harvested and placed in PBS solution for transfer to the OCT imaging facility for *ex vivo* analysis. Harvested samples were taken out from PBS and mounted on a glass slide to bring them under the objective of spectral domain OCT system (Telesto III, Thorlabs Inc.) to record OCT images.

## 3 Results and Discussion

### 3.1 Numerical Validation of Nanosensitive Multifractal Detrended Fluctuation Analysis in Synthetic Submicron Scale Volume Structures

**Fig. 2:**
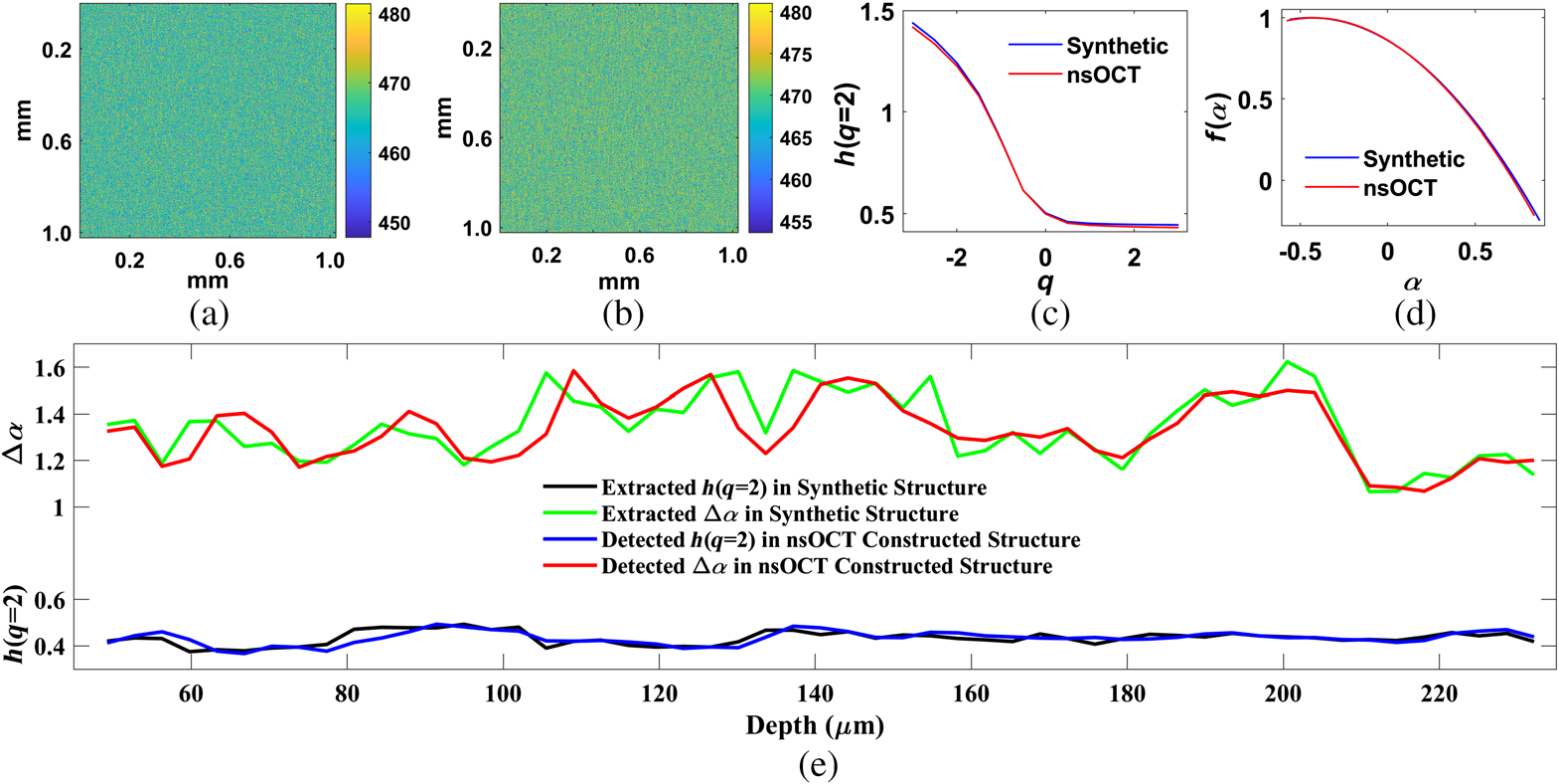
Detection of randomized submicron structural multifractality using nsMFDFA. (a) *En face* map of submicron synthetic structure at 150  μm depth. (b) *En face* map of submicron dominant structure at ∼150  μm depth extracted via nsOCT simulation. (c) Generalized Hurst exponents [h(q)] extracted at q=−3 to +3 from synthetic *en face* structure [blue color plot from Fig. 2(a)] and nsOCT detected *en face* structure [red color plot from Fig. 2(b)]. Extracted Hurst exponent, h(q=2)=0.45 from synthetic surface and h(q=2)=0.44 from nsOCT simulated surface. (d) Singularity spectrum [f(α) versus α) extracted from synthetic *en face* structure [blue color plot from Fig. 2(a)] and from nsOCT detected *en face* structure [red color plot from Fig. 2(b)]. Extracted width of singularity spectrum, Δα=1.43 from synthetic surface and Δα=1.42 from nsOCT simulated surface. (e) Depth-resolved self-similarity and multifractality. Depth-resolved Hurst exponents [h(q=2)] extracted from depth-resolved synthetic submicron scale *en face* structures (black color plot) and from nsOCT detected depth-resolved submicron scale *en face* structures (blue color plot). Depth-resolved width of singularity spectrum (Δα) extracted from synthetic submicron scale *en face* structures (green color plot) and from nsOCT detected submicron scale *en face* structures (red color plot).

We have recently demonstrated an experimental and numerical approach for nsOCT validation and detection of submicron structure with few nanometer accuracy.^19^ Here we have simulated nsOCT in synthesized volume (1024×1024×400  voxels) phantom composed of randomized submicron structures throughout the volume. Figure 2(a) displays synthesized *en face* map of submicron structure at ∼150  μm depth. Figure 2(b) displays corresponding nsOCT detected *en face* map of submicron structure. Figure 2(b) demonstrates that nsOCT can detect submicron scale dominant structure with 5-nm accuracy (comparing scale bar of synthetic submicron structure: 448 to 482 nm and nsOCT constructed scale bar: 443 to 482 nm). To check multifractality and correlation in synthesized *en face* and in nsOCT detected *en face*, we have applied state of the art 2D-MFDFA on each *en face* images throughout the depth. Figure 2(c) shows plots of generalized Hurst exponents [h(q) versus q] calculated from synthetic *en face* in Fig. 2(a) (blue color) and from nsOCT constructed *en face* in Fig. 2(b) (red color). Variation of h(q) versus q in Fig. 2(c) indicates that synthetic submicron structural *en face* has a multifractality (blue color plot) and can be detected with almost no error (red color plot). In addition, Hurst exponent [h(q=2)] in synthetic and nsOCT constructed *en face* is almost equal in values confirms our capability to detect submicron scale structural correlation within a complex tissue sample. Similarly, Fig. 2(d) shows plots of singularity spectrum [f(α) versus α] calculated from synthetic *en face* in Fig. 2(a) (blue color) and from nsOCT constructed *en face* in Fig. 2(b) (red color). The width of singularity spectrum (Δα) is a measure of multifractality is almost equal in synthetic and nsOCT extracted *en face* image proved our capability to detect submicron scale structural multifractality within a complex tissue sample.

The lower part of Fig. 2(e) displays 2D-MFDFA extracted depth-resolved Hurst exponent [h(q=2)] from synthetic (black color plot) and from nsOCT detected (blue color plot) submicron scale *en face* structures. The upper part of Fig. 2(e) displays 2D-MFDFA extracted depth-resolved width of singularity spectrum (Δα) from synthetic (green color plot) and from nsOCT detected (red color plot) submicron scale *en face* structures. Detected depth-dependent Hurst exponent [h(q=2)] and strength of multifractality (Δα) from synthetic and nsOCT simulated *en face* structure is very close to each other. These results validated our capability to detect submicron scale structural multifractality through the proposed nsMFDFA methodology from a tissue-like complex submicron scale multifractal structure with greater accuracy.

### 3.2 Application of Validated Nanosensitive Multifractal Analysis Technique on Pathologically Characterized MFP Tissue Samples

**Fig. 3:**
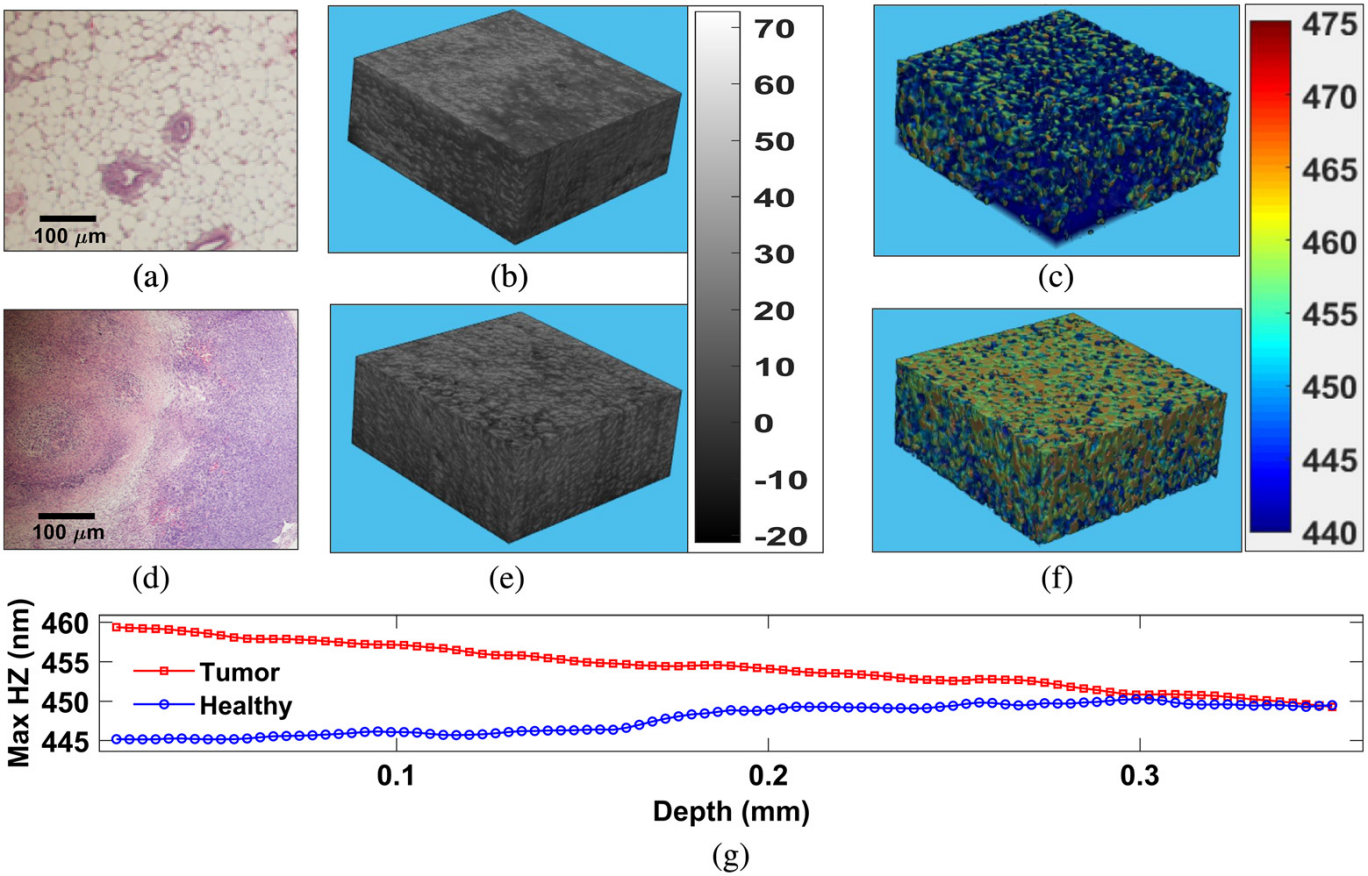
Hematoxylin and eosin (H&E) stained histological images (500  μm×500  μm) of (a) normal MFP and (d) tumor tissue. (b), (e) 3D volume (500  μm×500  μm×360  μm) OCT images of healthy and tumor tissue. Gray scale bar represents OCT intensity for Figs. 3(b) and 3(e). (e), (f) Dominant submicron axial structural mapped 3D volume OCT images of healthy and tumor tissue, respectively. Color bar represents size of dominant submicron axial structures for Figs. 3(c) and 3(f). (g) Depth-resolved nanosensitive structural averages at each *en face* for healthy MFP [blue plot extracted from volume in Fig. 3(c)] and tumor [red plot extracted from volume in Fig. 3(f)].

After successful validation of nsMFDFA approach, we have applied this technique on *ex vivo* tissue with healthy MFP and tumor portion. Figures 3(a) and 3(d) display hematoxylin and eosin (H&E) stained microscopic images of healthy MFP and tumor tissue, respectively. Figures 3(b) and 3(e) represent conventional OCT images from healthy MFP and tumor tissue, respectively. Note that average intensity values are 32.04 and 33.32, respectively, in healthy MFP and tumor tissue volume. Therefore, it is very difficult to differentiate OCT of healthy MFP and tumor tissue. Figures 3(c) and 3(f) display volume nsOCT where dominant axial submicron structure mapped onto healthy MFP and tumor tissue, respectively. Measured overall average maximum spatial period for healthy MFP is 447.5 nm and tumor tissue is around 454.6 nm. Although, we can see a difference in healthy and tumor tissue based on this overall volume average nanosensitive measurement, but they may not have differences at different depths. Figure 3(g) displays depth-resolved nanosensitive structural averages at each *en face* for healthy [blue plot extracted from volume in Fig. 3(c)] and tumor [red plot extracted from volume in Fig. 3(f)]. It shows that at 0.35 mm depth, the average value of dominant structure is almost the same for healthy and tumor tissue. Here it is difficult to differentiate tumor from healthy tissue based on local nsOCT measurement only. It is also unable to provide quantitative and depth-specific nanosensitive submicron scale tissue morphological complexities.

In these regards, it is known that tissues have multifractality in submicron structure. Therefore, it is always interesting to have depth-resolved quantitative submicron scale multifractal parameters for better understanding. Quantitative submicron scale multifractal parameters can also help to develop computer-assisted automated differentiation of tumor tissue from healthy tissue. In this direction, we have performed 2D-MFDFA on depth-resolved nsOCT constructed *en face* images to find nanosensitive multifractal parameters.

**Fig. 4:**
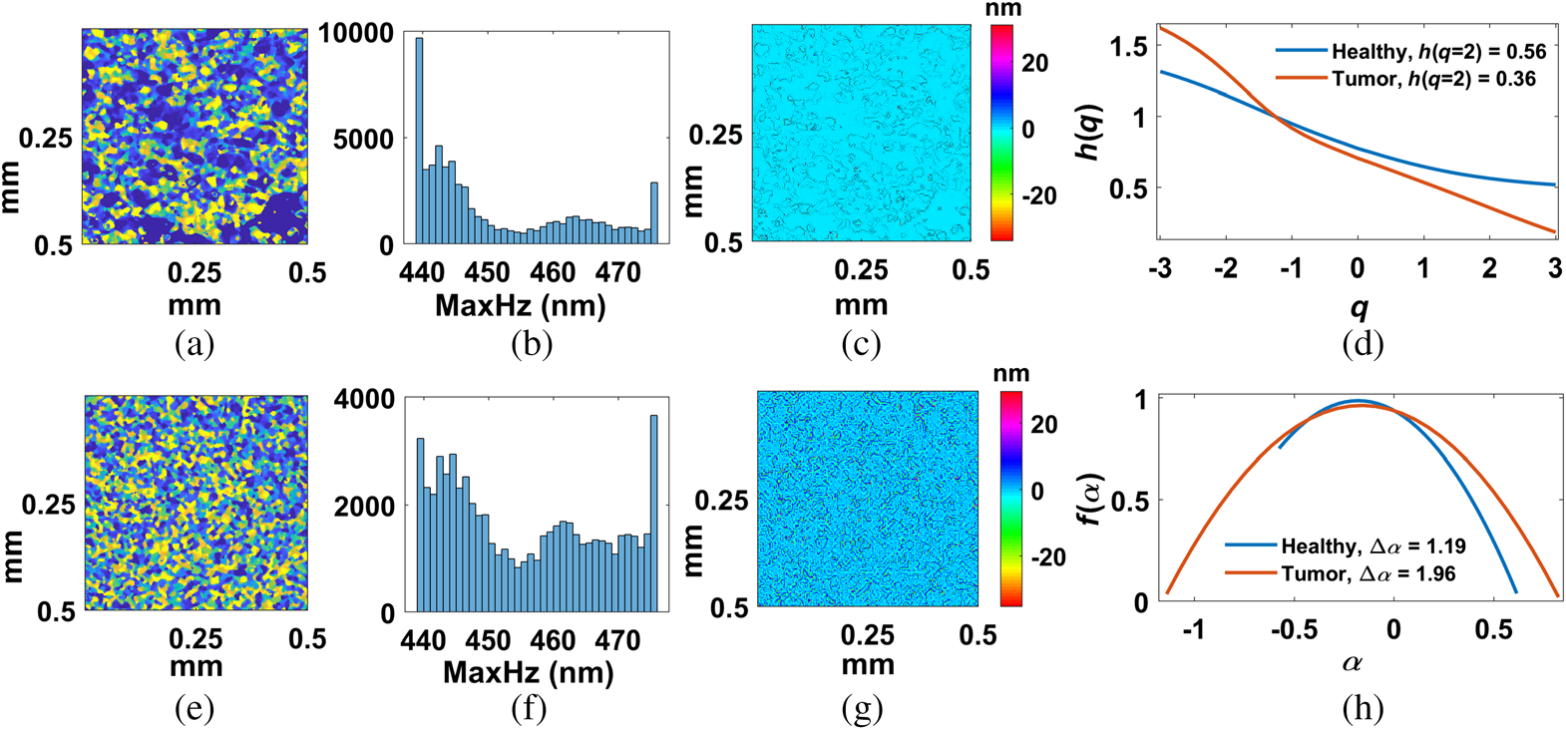
*En face* nsOCT image at 150  μm depth for (a) healthy and (e) tumor tissue corresponding to volume image in Figs. 3(c) and 3(f). Color bar represents size of dominant submicron axial structures. Panels (b) and (f) represent submicron structural histogram corresponding to Figs. 4(a) and 4(e). (c), (g) Corresponding detrended *en face* images with window size s=8  μm×8  μm. Color bar represents nanosensitive variation of local submicron axial structures. (d) Generalized Hurst exponent h(q) versus q for healthy (blue color) *en face* and tumor (red color) *en face*. (h) Singularity spectrum f(α) versus Holder exponent, α for healthy (blue color) *en face* and tumor (red color) *en face*.

In Figs. 4(a) and 4(e), we have displayed maximum spatial period *en face* images at 150  μm depth corresponding to volume images in Figs. 3(c) and 3(f). Figures 4(b) and 4(f) represent submicron structural histogram corresponding to Figs. 4(a) and 4(e). Figures 4(c) and 4(g) represent local nanoscale variation after subtracting local trends with detrending scaling window s=4 [see Eqs. (3) and (4)]. Figure 4(d) displays generalized Hurst exponent h(q) versus q for healthy (blue color) *en face* and tumor (red color) *en face* image. Figure 4(h) displays singularity spectrum f(α) versus Holder exponent, α for healthy MFP (blue color) *en face*, and tumor (red color) *en face* image. Variation of h(q) in Fig. 4(d) indicates multifractality and greater variation of h(q) indicates larger multifractality in tumor tissue [red color plot in Fig. 4(d)]. This multifractality reflected in singularity spectrum f(α) plots with larger width of singularity spectrum in case of tumor tissue [red color plot in Fig. 4(h)].

**Fig. 5:**
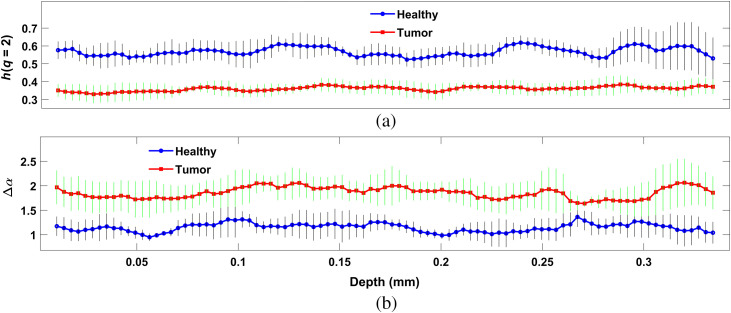
Overall multifractality in dominant submicron structure over *en face* images at different tissue depths at 10 different areas of the tissue sample. (a) Hurst exponent [h(q=2)] represents correlation of submicron structural distribution over *en face* for healthy (blue) and tumor (red) tissue at different depths. (b) The width of singularity spectrum (Δα) represents randomness of submicron structures over *en face* images at different depths for healthy (blue) and tumor (red) tissue. Vertical black lines, and vertical green lines are standard deviations at each depth.

For further verification and confirmation, we have applied this extraction method of nanosensitive multifractality on 10 healthy MFP and 10 tumor volume images in different areas of a tissue sample. We found consistence differences of nanosensitive correlation and strength of multifractality over different depths of tissue. Figures 5(a) and 5(b) represent mean Hurst exponent [h(q=2) using Eq. (5)] and strength of multifractality [Δα using Eq. (9)] over 10 healthy MFP (blue line plot) and tumor (red line plot) tissue on *en face* images at different depths. Vertical lines at each depth represent standard deviation from mean trends over 10 samples. In Fig. 5(a), reduction trends of nanosensitive Hurst exponent indicate decrease of correlation of dominant submicron structural distribution over *en face* images as tumor progress. In Fig. 5(b), increase of nanosensitive multifractality indicates increase of strength of multifractality or distortedness of dominant submicron structural distribution over *en face* images as tumor progress.

## 4 Conclusions

A novel approach to quantify submicron scale nanosensitive multifractality in combination with nsOCT and multifractal analysis has been demonstrated. We validated the nsOCT technique numerically on synthetic submicron scale axial structures. We developed a novel nanosensitive submicron scale multifractal analysis technique to characterize tissue depth-resolved ultrastructural morphology. Reduction of the Hurst exponent [h(q=2)] from healthy MFP to tumor indicates reduction of correlation or self-similarity in dominant submicron structures. Increase of width of singularity spectrum (Δα) from healthy MFP to tumor indicates increase of multifractality or roughness in dominant nanosensitive submicron scale tissue structures. This newly developed method promises early disease detection for better treatment guidance and monitoring response to treatment in cancer patients. Results promise to detect nanosensitive multifractality in the submicron scale structural distribution and its alteration in deep tissue as tumor progress. This ability to delineate nanosensitive self-similarity may provide a noninvasive measuring tool for characterization of biological tissue and nonbiological media. Observed differences in the submicron scale nanosensitive multifractality between healthy and tumor tissue show considerable promise as potential biomarkers for cancer detection. The ability to probe and quantify nanosensitive self-similarity and change of multifractality related to development of cancer using backscattering mode FD-OCT bodes well for *in vivo* deployment. Exploiting the interference spectra recorded from tissue depths with the reference mirror, *in vivo* applications of this approach should be realized with a fiber optic-based handheld probe assisted with scanning lens and galvo mirror. Finally, the developed nsMFDFA method represents as a novel approach with much potential for *in vivo* detection of cancer initiation and other non-biological application remain to be rigorously evaluated.
