# Characterization of nanosensitive multifractality in submicron scale tissue morphology and its alteration in tumor progression (Erratum)

**DOI:** 10.1117/1.JBO.26.1.019805

**Published:** 2021-01-30

**Authors:** Nandan Das, Sergey Alexandrov, Katie E. Gilligan, Róisín M. Dwyer, Rolf B. Saager, Nirmalya Ghosh, Martin Leahy

**Affiliations:** a National University of Ireland, Tissue Optics and Microcirculation Imaging, Galway, Ireland; b Linköping University, Biomedical Imaging and Spectroscopy, Clinical Instrument Translation, Linköping, Sweden; c National University of Ireland Galway, Discipline of Surgery, Lambe Institute for Translational Research, Galway, Ireland; d Indian Institute of Science Education and Research Kolkata, Bio-Optics and Nano-Photonics, Kolkata, India; e Institute of Photonic Sciences, Barcelona, Spain

## Abstract

An error in the 2nd author’s name is corrected.

This article [*J. Biomed. Opt.* **26**(1), 016003 (2021) doi: 10.1117/1.JBO.26.1.016003 was originally published on 11 January 2021 with an error in the name of the second author. Sergey Alexandrov was transposed as “Alexandrov Sergey.” The error was corrected on 19 January 2021.

